# Supplementary material for: Variations in kinase and effector signaling logic in a bacterial two component signaling network
Source: J Biol Chem. 2025 Apr 22;301(6):108534. doi: 10.1016/j.jbc.2025.108534 (PMC12148442; doi:10.1016/j.jbc.2025.108534)
Supplement: Supporting information [file mmc1.pdf]

Variations in kinase and effector signaling logic in a bacterial two component signaling network

Supporting Information

Danielle Swingle[1,2], Leah Epstein[1,2], Ramisha Aymon[1,3], Eta A. Isiorho[1], Rinat R. Abzalimov[1], Denize C. Favaro[1], Kevin H. Gardner[1,3,4*]

[1]: Structural Biology Initiative, CUNY Advanced Science Research Center, New York, NY 10031

[2]: Ph.D. Program in Biochemistry, The Graduate Center – City University of New York, New York, NY 10016

[3]: Department of Chemistry and Biochemistry, City College of New York, New York, NY 10031

[4]: Ph.D. Programs in Biochemistry, Biology, and Chemistry, The Graduate Center – City University of New York, New York, NY 10016

Contents:

- Supporting Table S1
- Supporting Figures S1-S5

Table S1: X-ray crystallography data collection and refinement statistics

	RT-PhyR (ruthe_01174)	RT-PhyR′ (ruthe_02744)
Data collection		
Space group	I 2 2 2	P 1 2_1 1
Unit cell parameters (Å)	*a* = 113.91	*a* = 60.101
	b = 118.642	*b* = 149.543
	c = 121.088	*c* = 73.564
X-ray source	17-ID-2 at NSLS-II	17-ID-1 at NSLS-II
Wavelength (Å)	0.979338	0.920105
Resolution range (Å)	34–1.985 (2.056–1.985)	33.85–2.825 (2.926–2.825)
Unique reflections	56823 (5587)	30306 (2907)
Multiplicity	2.0 (2.0)	2.0 (1.9)
Completeness (%)	99.92 (99.64)	99.33 (96.61)
Mean $I / \sigma(I)$	13.74 (0.96)	2.78 (0.68)
R_{meas}	0.03541 (1)	0.1792 (1.1)
$CC_{1/2}$	1 (0.459)	0.983 (0.38)
Refinement		
Reflections used in refinement	56818 (5587)	30305 (2907)
Reflections used for R-free	2675 (242)	1393 (149)
Number of non-hydrogen atoms	5975	7503
Macromolecules	5752	7458
Ligands	27	0
Solvent	208	45
R_{work}	0.2085 (0.3423)	0.2328 (0.3265)
R_{free}	0.2372 (0.3635)	0.2717 (0.3418)
RMS(bonds)	0.01	0.003
RMS(angles)	1.34	0.64
Protein residues	749	957
Average B-factor	54.23	59.09
Macromolecules	54.36	59.20
Ligands	53.28	
Solvent	50.72	41.04
Ramachandran plots		
Favored (%)	97.70	94.77
Allowed (%)	2.03	4.06
Outliers (%)	0.27	1.17
PDB accession code	9BY5	9CB6

Statistics for the highest-resolution shell are shown in parentheses.

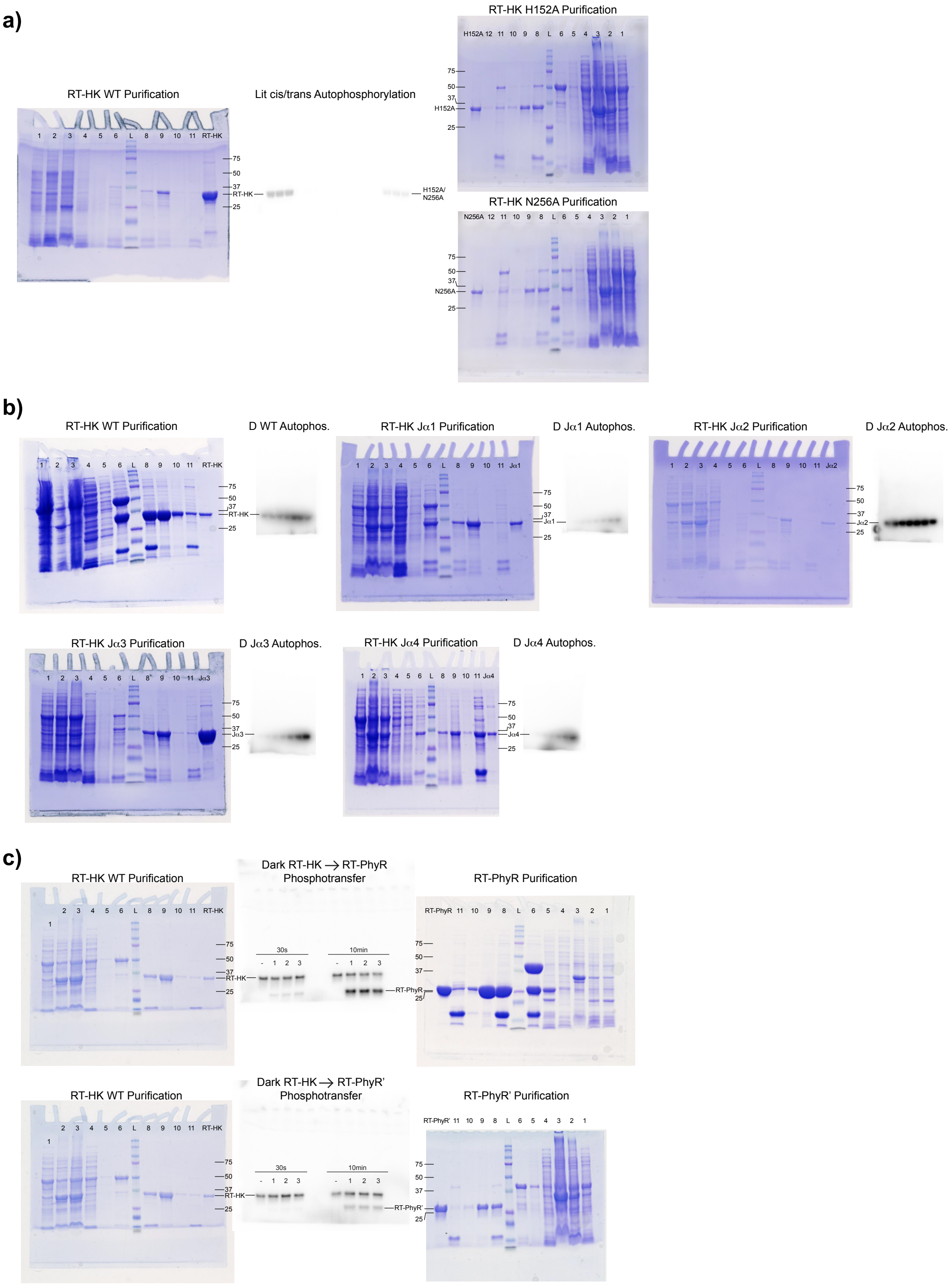

Figure S1: Purified protein samples used in ^{32}P radioactive assays (main text Figs 1, 3, 4). SDS-PAGE gels showing multiple purification steps (arbitrary numbers above lanes) used to arrive at the pure protein sample (protein name above lanes) used in each assay are shown alongside representative phosphorimages from: **a)** Cis/trans autophosphorylation assays (main Fig. 1f), **b)** Jα mutant autophosphorylation assays (main Fig. 3b), and **c)** Phosphotransfer assays (main Fig. 4b). Dehydrated gels were cut to remove free ^{32}P and ^{32}P-ATP signal prior to imaging.

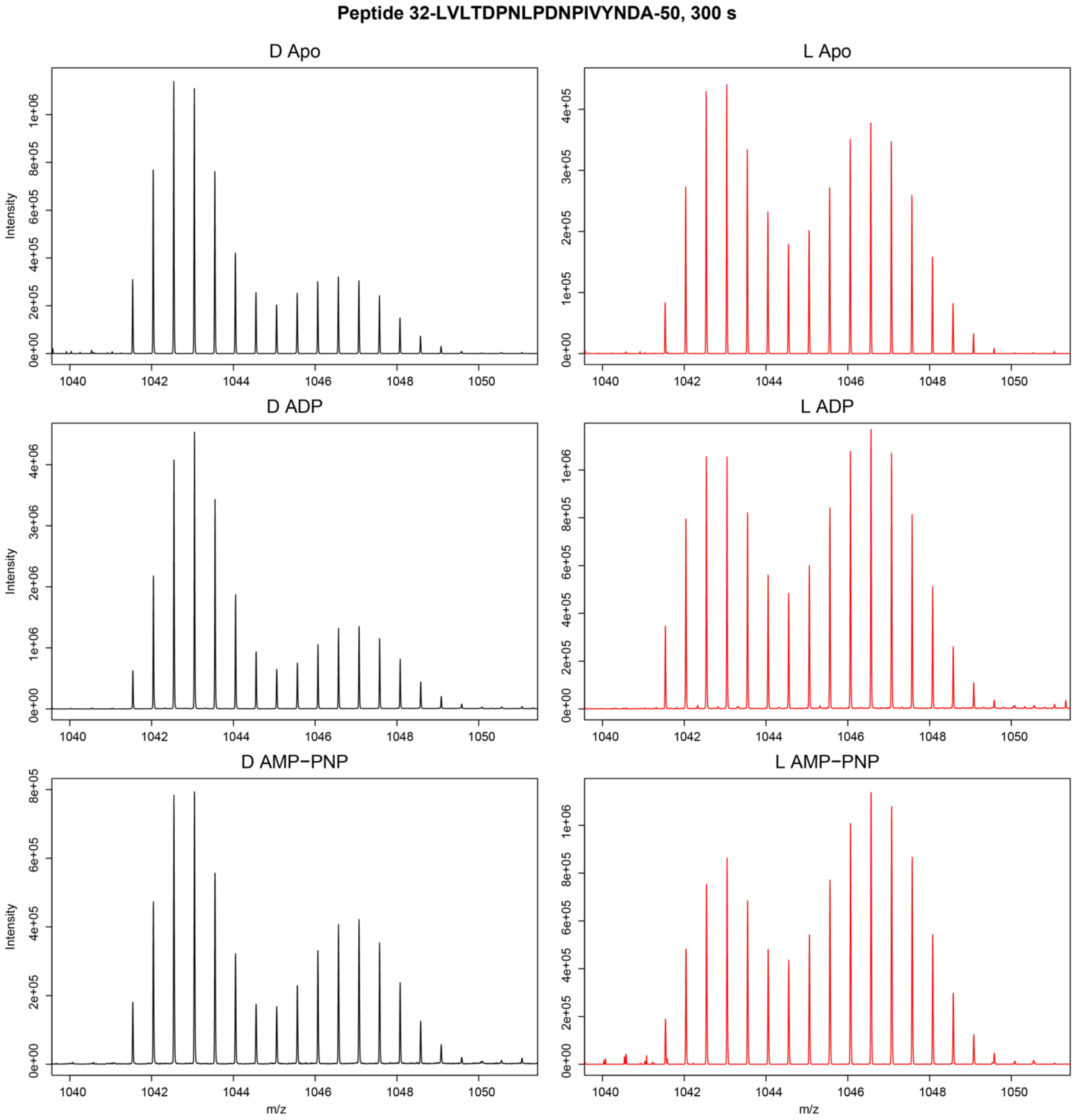

Figure S2: Lit state peptides have a relatively higher abundance of fast-exchanging population. A representative example of the bimodal distribution observed for peptides throughout the LOV domain is shown for residues 32-50 in all six protein states. The slow-exchanging population is represented by the left-most peak and fast-exchanging by the right peak. In the vast majority of cases, the fast-exchanging conformation was more abundant under lit conditions.

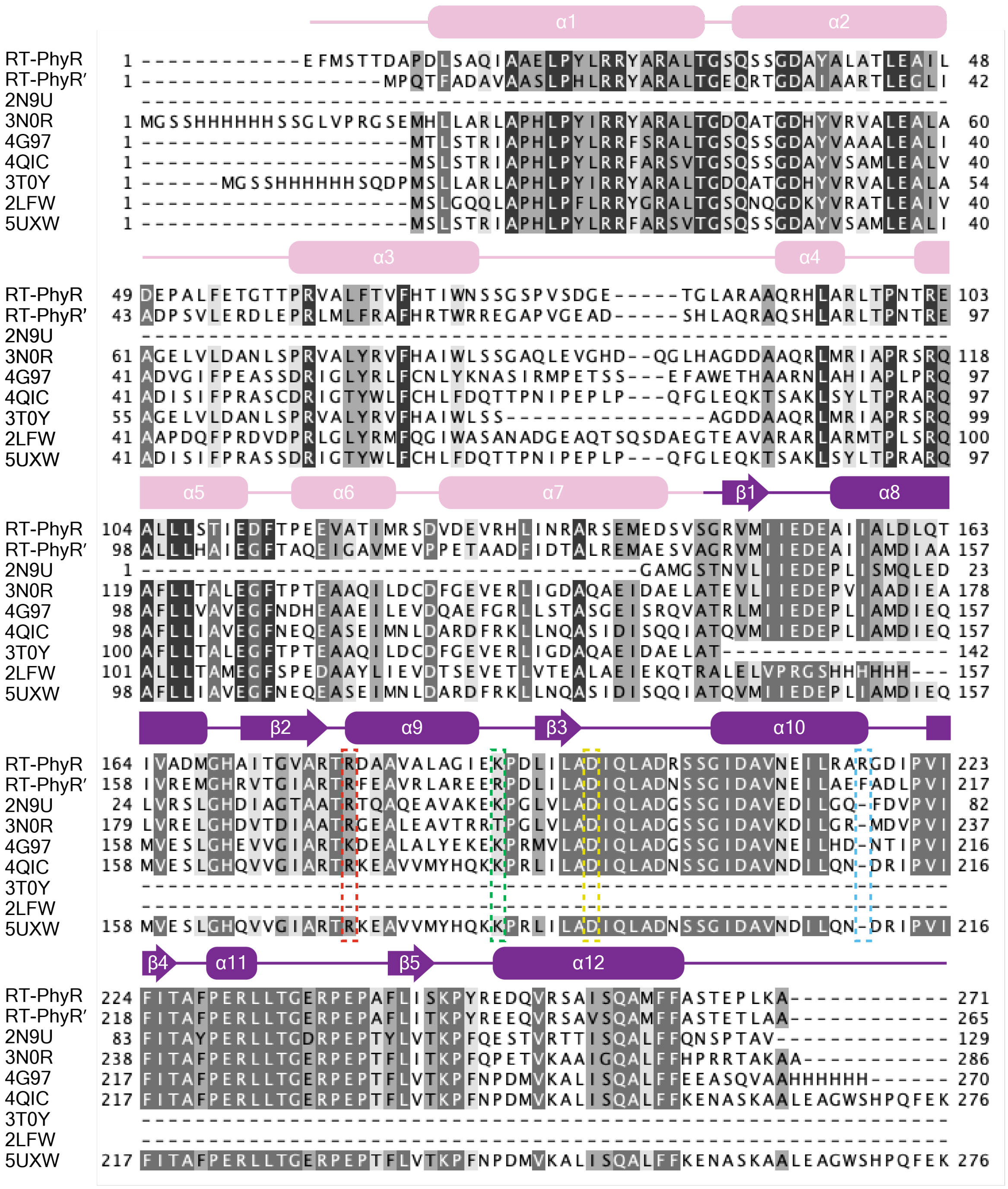

Figure S3: Multiple sequence alignment of RT-PhyR and RT-PhyR′ with other PhyRs of known structure. PDB IDs used as identifiers. Residues are shaded by consensus and thresholded at 50%. Secondary structure elements are depicted above each line, with SL domain colored in pink and REC in purple. Dashed boxes indicate important residues: yellow indicates the aspartate phosphorylation site, and red, green, and blue indicate sites defining RT-PhyR′ β3 loop–α10 helix shifts.

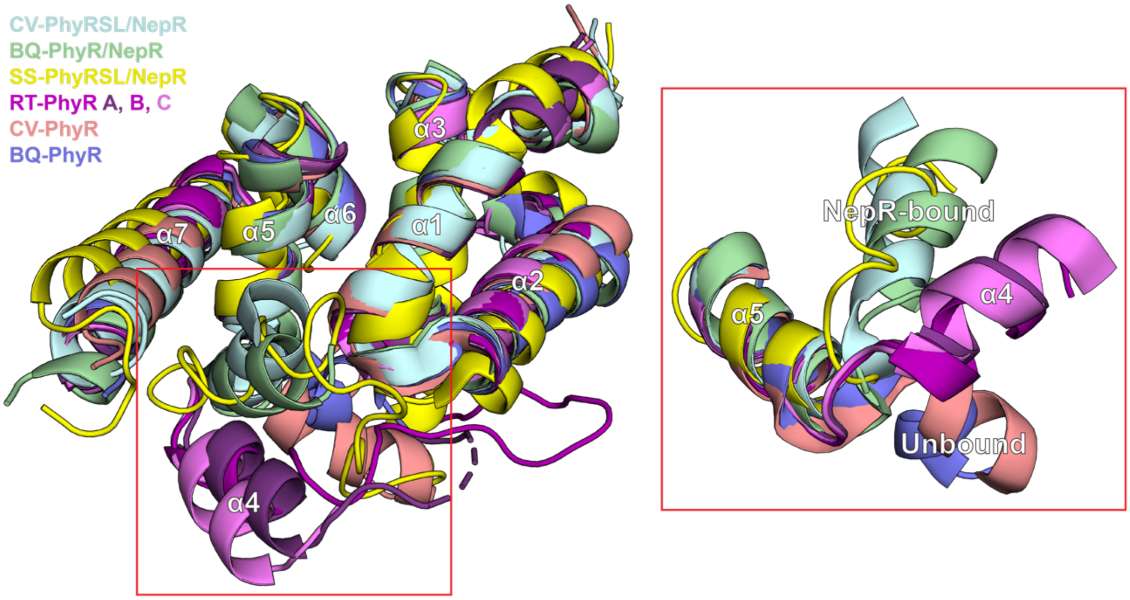

Figure S4: Position of RT-PhyR α4 differs from all other structures. Alignment of PhyR SL structures from other organisms with RT-PhyR SL (left). The RT-PhyR α4 helix does not adopt one of the positions defined for NepR-bound or unbound states (right). Structures are labeled according to first two letters of host organism— corresponding PDB IDs are 3T0Y, 4QIC, 2LFW, 9BY5, 3N0R, and 5UXW, from top to bottom. All chains from the RT-PhyR asymmetric unit are shown in different shades of purple, with slightly different α4 locations observed for each chain, which we attribute as likely to different crystal packing interactions with adjacent molecules in the asymmetric unit (chain A) or neighboring ones (chains B and C).

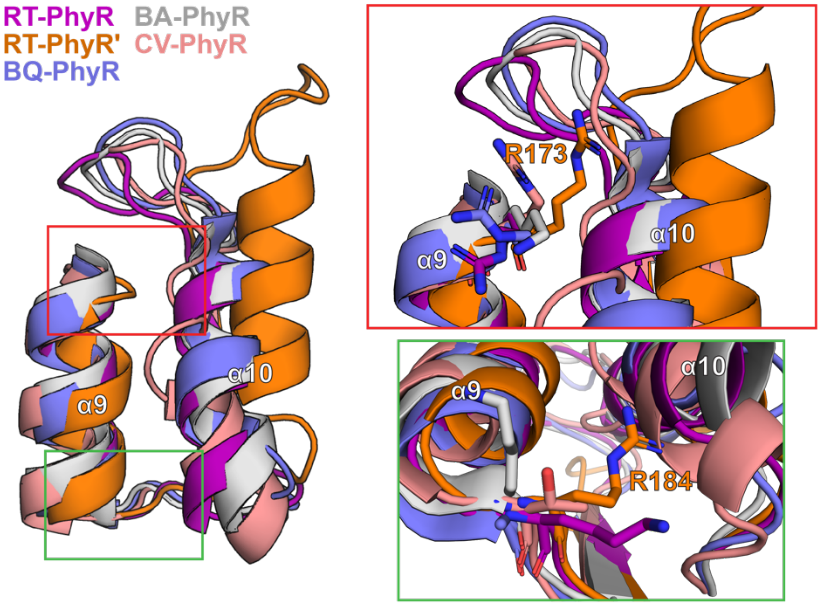

Figure S5: RT-PhyR′ residue position shifts in α9. Other known PhyR structures added to the alignment illustrate that the RT-PhyR′ β3 loop–α10 helix position is the outlier. The red zoomed in region highlights the shift in the position of R173 at the top of α9 and the green zoomed-in region shows the shift in R184 at the bottom of α9. Structures are labeled according to first two letters of host organism, with PDB IDs as follows: blue: 5UXW, grey: 4G97, and pink: 3N0R.
